# Application status and prospects of multimodal EEG-fMRI in HIV-associated neurocognitive disorders

**DOI:** 10.3389/fneur.2024.1479197

**Published:** 2024-12-05

**Authors:** Junzhuo Chen, Haixia Luo, Jiaojiao Liu, Wei Wang, Juming Ma, Chuanke Hou, Xingyuan Jiang, Zhongkai Zhou, Hongjun Li

**Affiliations:** ^1^Department of Radiology, Beijing Youan Hospital, Capital Medical University, Beijing, China; ^2^Department of Radiology, Qilu Hospital of Shandong University, Jinan, Shandong, China; ^3^Laboratory for Clinical Medicine, Capital Medical University, Beijing, China

**Keywords:** HAND, EEG, fMRI, simultaneous EEG-fMRI, multimodal imaging

## Abstract

HIV-associated neurocognitive disorders (HAND) are one of the common complications in people living with HIV (PLWH), which can affect their attention, working memory, and other related cognitive functions. With the widespread use of combination antiretroviral therapy (cART), the incidence of HAND has declined. However, HAND is still an important complication of HIV, which not only affects the quality of life of patients but also affects their adherence to HIV treatment. Its diagnosis mainly relies on neurocognitive tests, which have a certain degree of subjectivity, making it difficult to diagnose and classify HAND accurately, and there is an urgent need to explore more sensitive biomarkers. Multimodal brain imaging has seen a surge in recent years with simultaneous EEG-fMRI being at the forefront of cognitive multimodal neuroimaging. It is a complementary fusion technique that effectively combines the high spatial resolution of fMRI with the high temporal resolution of EEG, compensating for the shortcomings of a single technique and providing a new method for studying cognitive function. It is expected to reveal the underlying mechanisms of HAND and provide high spatiotemporal warning biomarkers of HAND, which will provide a new perspective for the early diagnosis and treatment of HAND and contribute to the improvement of patient prognosis.

## 1 Introduction

HIV-associated neurocognitive disorders (HAND) are a series of cognitive, behavioral, motor, and autonomic dysfunctions caused by human immunodeficiency virus (HIV) infection (1). HIV is neurophilic and invades the central nervous system (CNS) in the early stages of infection, causing brain damage and subsequently leading to HAND (2). With the widespread use of combination antiretroviral therapy (cART), the incidence of HAND has declined. However, HAND is still an important complication of HIV, which not only affects the quality of life of patients but also affects their adherence to HIV treatment (3, 4). The presence of HAND is associated with higher mortality, indicating the importance of early recognition and treatment of HAND (2).

At present, the diagnosis of HAND is mainly based on neurocognitive testing, but this method has a certain degree of subjectivity and cannot detect subtle neurocognitive changes. An increasing number of studies indicate that even without clinical symptoms or abnormal neurocognitive tests, people living with HIV still suffer from brain dysfunction (5, 6). Therefore, more sensitive methods are needed to assess potential neurobiological changes in people living with HIV during the asymptomatic phase.

Functional magnetic resonance imaging (fMRI) is a non-invasive brain imaging technique that can monitor changes in brain activity and functional connectivity during tasks or at rest. Through fMRI research, it has been identified that patients with HAND exhibit weakened functional connectivity and altered brain activity patterns in multiple brain regions, including the prefrontal cortex, temporal lobe, and hippocampus (7). Electroencephalogram (EEG), as another important brain function monitoring technique, provides high temporal resolution neurophysiological information by recording brain electrical activity. The EEG studies have revealed significant changes in neural electrical activity, event-related potential (ERP), and brainwave rhythms in patients with HAND, which are closely related to the degree of cognitive impairment (8). The fusion multimodal method of simultaneous EEG-fMRI effectively combines the high spatial resolution of fMRI and the high temporal resolution of EEG, making up for the shortcomings of a single technique. It links changes in brain blood oxygen levels with neuro electrophysiological information, potentially revealing high spatiotemporal warning biomarkers for HAND.

## 2 Overview of HAND

### 2.1 Performance of HAND

In the early stage of HAND, patients mainly exhibit attention deficits, impaired memory, executive function, etc. As the condition progresses, patients may experience consciousness disorders and mild motor disorders primarily characterized by emotional disorders such as depression. When it progresses to the late stage, if effective treatment is not obtained, dementia symptoms may occur, and it may also develop into a bedridden state accompanied by silence and urinary incontinence (9). Before cART, the most common manifestations of HAND were motor delays and slowed processing speed, while in the era of cART, learning, memory, and executive dysfunction were more common manifestations (10).

### 2.2 Stages of HAND

In 2007, the American Academy of Neurology classified HAND into three stages based on the Frascati criteria: asymptomatic neurocognitive impairment (ANI), mild neurocognitive disorder (MND), and HIV-associated dementia (HAD) (11). This criterion is based on neurocognitive testing and self-reported daily activities. However, there are also current studies suggesting that this classification method may overestimate HAND. The minimum criteria for HAND are based only on the performance of cognitive tests, which may not be suitable for people with different educational and socioeconomic backgrounds. Among them, although the ANI stage is asymptomatic, some evidence indicates that it may have clinical relevance while others support the view that this definition may lead to overdiagnosis of HAND and increase the burden on patients (3, 4). Nevertheless, HAND remains an important complication of HIV, exerting a profound impact on many aspects of an individual’s function and quality of life (12). The diagnostic and classification methods of HAND must reflect the modern spectrum of the disease so that the prognostic information is accurate and the affected individuals can obtain the required assistance (13). Therefore, it is necessary to further develop more perfect staging criteria for HAND in the future.

### 2.3 Mechanism of HAND

The mechanism of HAND is multifaceted, including direct and indirect factors, mainly including the following aspects:

(1) Destruction of the blood–brain barrier (BBB): In the early stage of infection, HIV mainly crosses the BBB into the CNS through infected lymphocytes and monocytes, and HIV can damage the structure, function, and multicellular components of the BBB, leading to increased permeability of the BBB (14). This change allows more viruses and inflammatory cells to enter the brain, exacerbating inflammation and damage to the brain.
(2) Neurotoxic effects: Although neuronal cell surfaces are not susceptible to HIV-1 infection due to the lack of CD4 receptors, HIV can promote inflammation or have direct toxic effects on neurons through neurotoxic proteins released by monocytes/macrophages and glial cells, such as gp120, Tat, Vpr, etc. (2, 14). These viral proteins cause direct neurological damage by binding to cell surface receptors and interfering with normal neuronal function, leading to cell dysfunction or death (15).
(3) Immune activation and inflammatory response: Chronic immune activation and inflammatory response caused by HIV infection also play an important role in the development of HAND. HIV infection activates microglia, astrocytes, and macrophages, releasing inflammatory factors (e.g., TNF-*α*, IL-1β) and neurotoxic substances (e.g., ATP, arachidonic acid), which can damage neurons and neuronal support cells, leading to an imbalance in central nervous system homeostasis and subsequently affecting brain function (16). In addition, even in the era of cART, due to the limited ability of cART drugs to penetrate the BBB, it may not be able to eliminate the HIV reservoir in the brain (1). Low levels of HIV persist in the CNS, leading to sustained low-level inflammatory responses in neurons, chronic axonal damage, demyelination, and ultimately neuronal degeneration and death (17, 18).
(4) Neurotoxicity of cART: Although cART significantly improves the survival rate of people living with HIV and reduces the viral load of HIV in plasma, cART may also induce neurotoxicity, leading to cognitive impairment (19). For example, efavirenz (EFV) has a wide range of neuropsychiatric side effects that can cause insomnia, excessive dreaming, and other symptoms (20). Studies showed that patients with EFV-containing cART have worse cognition than those on other drugs, and cognition improves after EFV is discontinued (10, 21).
(5) Metabolic disorders: HIV infection is also associated with metabolic disorders in the brain, including mitochondrial dysfunction, abnormal lipid metabolism, increased oxidative stress, etc., all of which may lead to neuronal damage (14).

## 3 Application of fMRI in HAND

### 3.1 Technical principles, advantages, and disadvantages of fMRI

fMRI is a noninvasive functional neuroimaging technique used to observe the functional activity of the brain during different tasks or resting states. The basis of fMRI is the blood-oxygen-level dependent (BOLD) signal, which reflects changes in cerebral blood flow and oxygenated hemoglobin concentration. When a brain region is active, local blood flow increases, enhancing BOLD signals in that area. This is because active neurons require more oxygen and nutrients (22). fMRI can be used to study the functional connections between different brain regions, revealing the collaborative work of different brain regions during task performance or resting state. It provides high spatial resolution and can detect small functional areas in the brain, which is very advantageous in locating task-related activities and abnormal regions. It generates images with high contrast, allowing researchers to visualize the spatial distribution of brain activity, but the temporal resolution of fMRI is low. BOLD signals change slowly, failing to capture details of fast neural activity (23).

### 3.2 Application of rs-fMRI in HAND

Resting-state functional magnetic resonance imaging (rs-fMRI) is simple to operate and reproducible relative to task-state fMRI, avoiding the interference of task design, environment, and other factors, and has been widely used for mechanism research, early diagnosis, and treatment evaluation of HAND.

There are three commonly used analytical metrics for rs-fMRI:

(1) Regional homogeneity (ReHo): It refers to the synchronization between the local activity of a voxel and the activity of neighboring voxels, mainly reflecting the temporal consistency and synchronization of the blood oxygen level in the local brain area (24). Bak et al. (25) compared ReHo values in three groups: HAND, HIV patients with intact cognition (HIV-IC), and healthy controls (HC). Compared with HC, HAND displayed a wider range of ReHo value anomalies. Compared with HIV-IC, HAND had a higher ReHo value in the right primary sensory-motor area. HAND had more damage to the orbitofrontal cortex and primary sensory motor area than HIV-IC, and was associated with behavioral performance. Han et al. (26) evaluated the correlation between ReHo and early cognitive function in HAND, and the results showed that multiple regions of the visual network in ANI patients had functional abnormalities. The decrease in ReHo values, represented by the right lingual gyrus, indicated a decline in the encoding and logical analysis of visual memory in ANI patients.
(2) Amplitude of low-frequency fluctuations (ALFF): ALFF reflects the average voxel amplitude of the brain in the low-frequency range (0.01–0.08 Hz), reflecting the local spontaneous activity of the brain at rest (27). Bak et al. (25) found that compared with HC, HIV-IC, and HAND had lower ALFF in both frontal lobes, while bilateral occipital ALFF was higher. There was no significant difference in ALFF between the HAND and HIV-IC groups. Li et al. (28) showed that compared with HC, PLWH exhibited higher ALFF in the caudate nucleus and frontoparietal cortex. The ALFF values in the auditory cortex were higher in PLWH who received cART than those who did not receive cART.
(3) Functional connectivity (FC): FC refers to the time-series coefficients between a certain subset of voxels and other voxels or regions of interest in the brain, mainly evaluating the changes in functional collaboration between two different brain regions. Multiple rs-fMRI studies have shown that FC changes occur in PLWH (29, 30). Wang et al. (29) found significant FC abnormalities between the cerebrum and cerebellum in patients with ANI, and the FC intensity between the right cerebellum and the left anterior cingulate cortex was negatively correlated with attention/working memory scores. Chaganti et al. (30) showed that HAND was significantly associated with reduced connectivity in Default Mode Network (DMN), Executive Control Network (ECN), and Salience Network (SAN).

All of the above demonstrated that Reho, ALFF, and FC may be potential biomarkers for HAND. In view of the above research, fMRI will also play an important role in the graded diagnosis of the severity of HAND in the future.

### 3.3 Application of ts-fMRI in HAND

Task-state fMRI (ts-fMRI) analyzes functional regions through real-time dynamic activation of the brain during the performance of specific tasks, thereby linking neuroimaging information to behavioral performance and identifying neuroanatomical regions of specific cognitive impairment (31). The fluctuation of BOLD response in specific brain regions indirectly reveals the coupling between neuronal activity and changes in cerebral blood flow under specific stimuli, and the increase or decrease in brain activity during task execution is believed to be related to the cognitive function being performed (6, 32). In recent years, this advanced neuroimaging technique has shown its effectiveness in studying the cognitive function and structural changes of the brain in HAND (6).

Neuropsychological research suggests that areas of the brain involved in attention, working memory, and situational memory may be particularly susceptible in people with HAND (5, 33, 34). In simple attention tasks, the parietal lobe has greater activity in PLWH, while in more complex attention tasks, the frontal and temporal lobes have greater activity (35). Other studies have shown that when PLWH perform visual attention tasks, the activation of the normal visual attention network decreases, while the activation of adjacent or contralateral brain regions increases. This suggested that PLWH need to restructure and increase the use of neural reserves to maintain task performance similar to the normal group. Exceeding the brain reserve capacity may lead to attention deficit and cognitive impairment (36). Similarly, in situational memory encoding tasks, although the activation of the hippocampal-prefrontal regions is weakened in PLWH, they exhibit additional activation of the lateral and posterior parietal lobes, which may also be a compensatory activation (37).

Activation of the lateral prefrontal cortex and parietal region increased in PLWH during working memory tasks (35, 38). Compared with HC, PLWH had more activation on simple tasks with similar accuracy and less activation on difficult tasks with decreased accuracy (5). In addition, it has been shown that the basal ganglia region exhibits increased activation in the early stages of HIV infection, which decreases as the disease progresses until it falls below normal levels (39). This increased activation of brain regions early in HIV infection and simple tasks reflects a compensatory mechanism, and when the compensatory capacity exceeds a certain level, a loss-of-compensation response such as decreased activation and poorer task performance occurs. This suggests that exceeding the brain’s reserve capacity may lead to HAND. This compensatory mechanism occurs in the context of sustained inflammatory response and glial cell proliferation caused by HIV, and neuronal remodeling often occurs in specific pathways such as attention/working memory, visual–spatial ability, delayed recall, and language recognition in related brain regions. This may be closely related to the functional reorganization caused by the brain injury (40).

In addition, longitudinal studies have shown that the task performances of PLWH before and after two occasions were similar to HC, but the fMRI signals in the prefrontal and posterior parietal cortex of PLWH were significantly activated (41). This indicates that PLWH maintain task state performance by increasing the use of attention networks, but their decreased neural efficiency indirectly reflects the sustained brain damage caused by HIV infection. These findings also indicate that PLWH may still develop to HAND even under sustained cART and virus control.

## 4 Application of EEG in HAND

### 4.1 Technical principles, advantages, and disadvantages of EEG

The cerebral cortex is composed of a large number of neurons that are interconnected through synapses and transmit electrical signals. When a large number of neurons discharge synchronously, potential differences are generated, which can be detected by electrodes on the scalp (42). EEG is a technique that records the electrical activity of brain neurons by placing electrodes on the scalp, primarily detecting changes in postsynaptic potentials of cortical neurons to reflect the functional status of the brain. Synchronized discharges of neurons are classified as spontaneous EEG and evoked EEG. Spontaneous EEG activity persists in the living brain and is usually categorized by frequency, including delta waves (2–4 Hz), theta waves (4–8 Hz), alpha waves (8–13 Hz), beta waves (13–30 Hz), etc. Different frequency bands represent various states and activities of the brain, which are associated with cognitive and emotional states (43). ERP is a special form of EEG that records the brain’s electrophysiological response to a specific event or stimulus. ERP usually appears within 100–500 milliseconds after stimulus onset and is closely related to cognitive processes such as attention, memory, and decision-making (44). ERP consists of different waves, each associated with a specific cognitive process, such as P300 related to the allocation of attention resources and stimulus assessment, and N100 related to auditory stimuli and language processing. Real-time monitoring of neuronal electrical activity by EEG and ERP with high temporal resolution up to the millisecond level. However, due to the electrode placement on the scalp surface and the volume conduction effect of structures such as skull and cerebrospinal fluid, EEG cannot accurately locate the activity of deep brain structures, resulting in low spatial resolution (45).

### 4.2 Main applications of EEG and ERP in HAND

PLWH are characterized by diffuse abnormal resting EEG rhythms, which are associated with cognitive impairment. Previous studies have shown that PLWH have increased frontal and central parietal delta power at rest and decreased alpha power preceded neurocognitive impairment. Alpha rhythms are the most important neural oscillatory activity, dominant in vision, hearing, and somatic movement. In addition, low-frequency alpha sources in the parietal, occipital, and temporal lobes are positively correlated with memory function (46, 47). Compared with HC, the amplitude of theta, alpha, and beta bands in the HIV group receiving cART generally decreased. In the untreated HIV group, the decrease in alpha band amplitude was more significant than in the cART group (47). In untreated HIV-infected individuals, there was a correlation between resting state EEG variables and CD4 counts, revealing that the synchrony of EEG production by cortical neurons was influenced by the impact of HIV on the immune system (46).

Studies have shown that ERP can reveal early electrical activity abnormalities in PLWH before clinical evaluation or structural MRI reveals brain abnormalities (48, 49). P300 has a long history of detecting the neurophysiological effects of HIV (49–52). P300 occurs approximately 300–500 ms after stimulation, reflecting the activation of the attention system and the allocation of attention resources related to orienting and working memory (53). ERP studies on neurophysiological abnormalities in PLWH have mostly been revealed by attention and working memory tasks (49, 54–56). When performing auditory or visual oddball tasks, PLWH have smaller P300 amplitudes and slightly longer latency periods. What’s more, the decreased P300 amplitude was positively correlated with the severity of HIV (minimum CD4 count), and the prolonged P300 latency was positively correlated with the increased HIV viral load. The difference in P300 was consistent with cognitive decline. The decreased P300 amplitude indicated a reduction in available attention resources during HIV infection, while the prolonged P300 latency indicated processing speed was affected (57). All of the above indicated that P300 can be used as a biomarker for HAND. Meghdadi et al. (8) used more complex sustained, focused and distracted attention tasks to help detect attention defects unique to HAND. Compared with the HC group, the HIV group showed smaller late positive potentials (LPP) and larger P200 amplitudes in all tasks, and the increase in P200 amplitude was positively correlated with the duration of HIV infection.

In addition, there have been reports of decreased amplitude and increased latency of P100, N100, N200, and N400. However, the group effect of these components was not as strong as that of P300, and the variables that most reliably differentiated PLWH from HC were still alpha power and P300 amplitude (48).

## 5 Comprehensive application of simultaneous EEG-fMRI

EEG and fMRI provide complementary advantages in recording temporal and spatial resolution of brain activity. fMRI can provide spatial localization of brain activity with millimeter-level accuracy, however, the BOLD response is too slow to capture the temporal dynamics of brain activity adequately. In contrast, EEG signals are directly coupled with the electrical activity of neurons with millisecond precision, capturing dynamic changes in brain activity. However, EEG cortical activity can only provide limited spatial resolution due to ambiguous spatial mixing. Therefore, each technique, when used in isolation, does not reflect the many-to-many mapping between activated brain regions and specific time points (23). Simultaneous EEG-fMRI technology combines the high temporal resolution of EEG and the high spatial resolution of fMRI to synchronize the recording of both electrophysiological and oxygen metabolism signals of brain activity, providing a high spatiotemporal resolution that individual methods cannot achieve.

**Figure 1:**
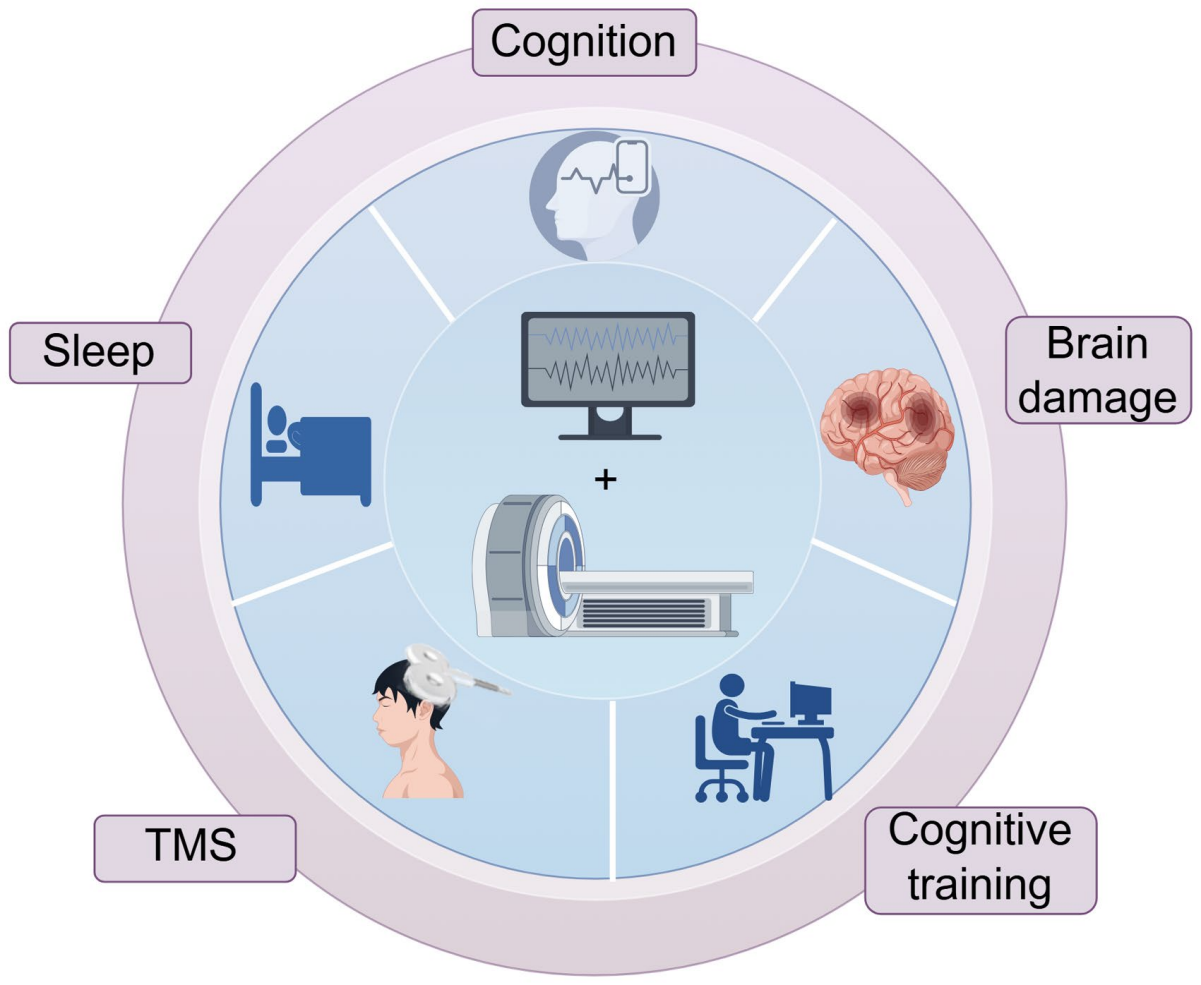
Application of simultaneous EEG-fMRI technology.

In recent years, simultaneous EEG-fMRI technology has been gradually maturing (22, 58). Simultaneous acquisition is the recording of electrophysiological activity at the same time as fMRI scan is performed, which ensures that the two modal data are acquired under conditions of identical neural activity (e.g., identical sensory stimuli and identical psychological and physiological states, behavioral responses, etc.) and that EEG and fMRI signals are directly time-correlated. It has been widely used in cognitive function studies, sleep research, mechanisms of brain injury in various neurological disorders, and assessment of the efficacy of related interventions (Figure 1).

### 5.1 Comprehensive application of simultaneous EEG-fMRI in the nervous system

#### 5.1.1 Simultaneous EEG-fMRI in Alzheimer’s disease

Previous studies have shown that as people progress from healthy aging to amnestic mild cognitive impairment (aMCI) and then to Alzheimer’s disease (AD), the whole-brain resting-state alpha rhythm gradually decreases. The alpha power decrease and theta rhythm increase may serve as markers of AD (59). P300 is related to cognition and memory and is the most widely used ERP component in studying dementia and aging. The changes in P300 can potentially differentiate between HC, MCI, and AD patients (60). In patients with MCI/AD, fMRI mostly shows weakened activation in brain regions, and a few brain regions show hyperactivity, which may be a compensatory effect. EEG and fMRI are important tools for revealing functional brain changes in AD. Still, a single technique is not sufficient to fully reveal the potential pathological changes in the continuous stages of AD. Combining the two heterogeneous data sources of EEG and fMRI is beneficial for improving the accuracy of diagnosis and differential diagnosis of AD (61). Simultaneous EEG-fMRI can identify early brain dysfunction from spatial and temporal dimensions, showing significant advantages in the early detection of possible AD markers.

AD is also one of the most common causes of cognitive impairment. Cognitive function relies on complex spatiotemporal neural dynamics, with many brain regions contributing at various time points. Simultaneous EEG-fMRI can achieve many-to-many mapping between brain regions and time points, accurately locate activated brain areas during cognitive processes, and determine the dynamic processes of neural activity (23). In recent years, simultaneous EEG-fMRI studies have been applied to classical cognitive tasks. Shu et al. (62) compared the spatiotemporal characteristics of healthy elderly subjects and patients with aMCI in a situational retrieval mode using the simultaneous task-state EEG-fMRI technique and found that although patients with aMCI and healthy controls had similar activation patterns of retrieval success, the electrophysiological activity of the recall and postretrieval monitoring processes was significantly weakened in patients with aMCI. The changes in the fMRI correlations of the ERP recall components were related to poorer memory performance, indicating that this technique is helpful for the early identification of individuals at high risk of cognitive deterioration. Cecchetti et al. (63) used fMRI network-driven EEG analysis to study and found that there were changes in the indicators related to network efficiency in AD dementia (ADD) patients in the theta band, and the relevant indicators of the right frontoparietal network in ADD patients decreased in the alpha2 band. MCI is between ADD patients and healthy individuals. The integration of EEG/fMRI highlights the role of the alpha2 frequency band as a potential biomarker of neurodegeneration.

Dementia differentiation is highly related to HAND, because in the aging PLWH population, other factors can lead to cognitive impairments via distinct neuronal mechanisms, such as vascular risks, AD, etc. (54, 64). AD and HAND share certain similarities in mechanisms of cognitive impairment and neuropathology. They are all likely to have memory loss, impaired executive function, and reduced language performance (65, 66). Neuropathologically, there are all abnormal protein accumulations (related to Aβ and Tau proteins), genetic factors (e.g., ApoEε4), metabolic disorders, blood–brain barrier damage, and changes in cerebrospinal fluid markers (67–69). They all have neuroinflammation involving inflammatory cell activation and inflammatory mediator release, all of which can lead to neuronal damage and loss, affecting cognitive functions (67, 70, 71). Although there have been no simultaneous EEG-fMRI studies applied to HAND, the technique has provided the basis for early diagnosis, intervention, and treatment of AD. Therefore, this technology also has a promising application in HAND, including early diagnosis and staging, evaluation of treatment efficacy, and in-depth study of the pathogenesis of HAND.

#### 5.1.2 Simultaneous EEG-fMRI in other neurological disorders

In addition, simultaneous EEG-fMRI has also been applied in other aspects of the nervous system. For example, Li et al. (72) discovered through the simultaneous EEG-fMRI technique that the connection of the anterior insula (aINS)-cortical network in patients with insomnia disorder was increased during wakefulness and non-rapid eye movement sleep (NREM), providing new evidence for the hyperarousal and inhibitory deficits of insomnia. Simultaneous EEG-fMRI technology assists in the precise localization of epileptic foci, preoperative electrode placement, guiding surgical resection, and therapeutic interventions, especially for the detection of epileptic activity with occult foci or structural MRI negativity. It can identify fMRI signals associated with epileptiform discharges in the interictal period, thus determining the origin and conduction process of epileptiform discharges (73). In a study testing whether alpha event-related desynchronization (alpha ERD) in children with attention deficit hyperactivity disorder (ADHD) is related to frontal parietal occipital connectivity, two key differences were found between ADHD children and their peers: the relationship between alpha ERD and occipital activity was weak, but the enhanced connectivity within the frontoparietal-occipital network during alpha ERD may reflect a compensatory mechanism and correlate with inattentive symptoms (74).

#### 5.1.3 Simultaneous EEG-fMRI in the assessment of intervention efficacy

Simultaneous EEG-fMRI technology can also assess the intervention effects of transcranial magnetic stimulation (TMS), computerized cognitive training, and other interventions on the brain. This technology provides both metabolic and electrophysiological evidence for TMS intervention effectiveness. It can evaluate the effect of TMS on specific brain regions based on its impact on spatiotemporal network dynamics, which helps to establish a causal relationship between the spatiotemporal recognition of neural responses and cognitive functions (22, 23). It can also be used to evaluate and track the effectiveness of brain rehabilitation techniques such as computerized cognitive training and neurofeedback training for specific brain regions (75).

### 5.2 Fusion analysis methods of simultaneous EEG-fMRI

**Figure 2:**
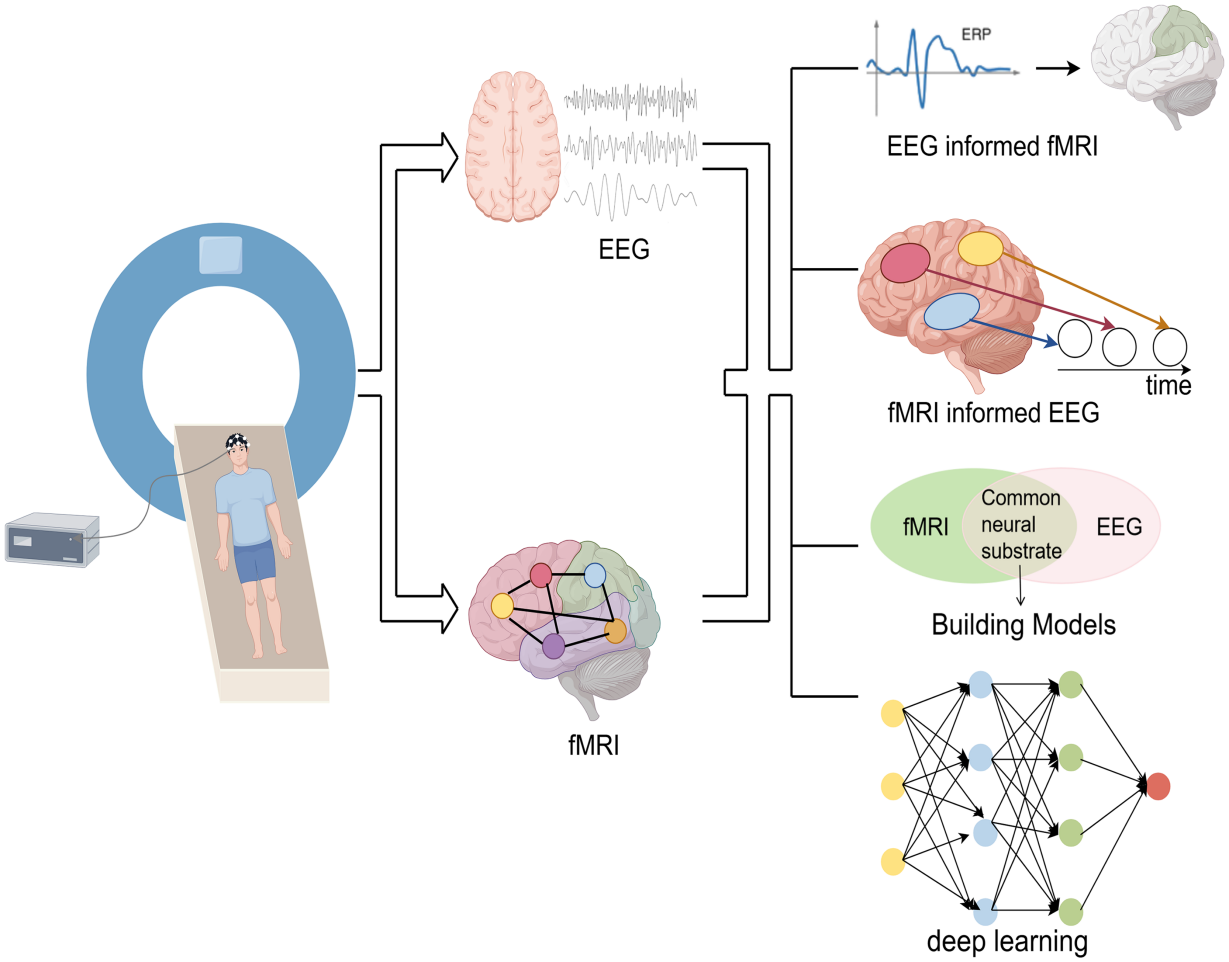
Simultaneous EEG-fMRI fusion analysis methods.

The fusion analysis methods of simultaneous EEG-fMRI are mainly divided into asymmetric fusion and symmetric fusion. The asymmetric methods use information from one modality to predict or constrain another modality. Symmetric fusion methods combine EEG and fMRI information in a balanced manner, avoiding potential biases in common neural activity inference caused by imbalanced use of modal information (76). The main fusion analysis methods are shown in Figure 2.

Asymmetric fusion methods include the following two types:

(1) EEG-informed fMRI: This method is currently the most widely used. It extracts EEG features of interest (e.g., ERP amplitude, latency, power, etc.), convolves them with fMRI hemodynamic response function (HRF), and incorporates EEG features as predictors of BOLD signals into the general linear model (GLM) analysis of fMRI to identify activated brain regions related to EEG features (77).
(2) fMRI-informed EEG: fMRI activation maps can be used as spatial constraints for EEG source localization. By utilizing the spatial information of fMRI to reconstruct the distribution of EEG sources or as spatial prior information, the accuracy of EEG source localization has been improved (76).

Symmetric fusion methods include the following two types:

(1) Model-driven approach: This method models the neurovascular coupling dynamics by identifying the common neural processes that trigger EEG and fMRI signals, and integrating them into a generative framework (22).
(2) Data-driven approach: This method avoids the complex modeling of EEG and fMRI generation processes, without considering the potential differences in their biophysical properties. This method utilizes the covariance between the two in the feature space and employs algorithms such as Independent Component Analysis (ICA), information theory frameworks, or machine learning (22, 76). For example, in ICA-based EEG-fMRI fusion, EEG temporal information and fMRI spatial information are connected into a single feature matrix and subjected to joint ICA decomposition, while generating spatially and temporally independent components (77). Recently, deep learning has provided new approaches for fusing EEG-fMRI. For example, Convolutional Neural Networks (CNN) implement temporal and/or spatial convolution operators in a form similar to linearized forward models that map neural sources into the space of EEG and fMRI measurements. The advantage of CNN lies in its ability to learn nonlinear representations, connecting two modalities and providing flexibility in mapping (76). Liu et al. (78) used CNN to map from one modality to another and showed that this method can learn the potential source space at EEG temporal resolution with a spatial resolution close to fMRI.

### 5.3 Limitations and challenges of simultaneous EEG-fMRI

Although simultaneous EEG-fMRI technology has significant advantages, it also faces many challenges in practical applications. The comprehensive benefits of simultaneous acquisition of EEG-fMRI come at the cost of reduced signal-to-noise ratio. The mutual interference between the two machines during simultaneous acquisition, poor comfort of the subjects, and the complexity of data fusion all pose difficulties in the implementation of simultaneous EEG-fMRI (22, 76, 79). In addition, another limitation of EEG-fMRI fusion is that it can only display parts of neural activity that are sensitive to both techniques, which may display relatively less temporal and spatial information than analyzing fMRI or EEG separately (23). In the future, the above problems may be solved through algorithm improvement, hardware upgrades, exploration of new data analysis methods, etc.

## 6 Conclusion and prospect

Simultaneous EEG-fMRI, as an advanced multimodal fusion technology, has been applied in clinical neuroscience. However, it has not yet been widely used in HAND research. Since there is a certain degree of subjectivity in the current diagnosis of HAND through neurocognitive scales, what’s more, a single-mode neuroimaging technique is not sufficient to fully reveal the potential pathological changes in the continuous stages of HAND. Future research will explore the application of this technology in the diagnosis and monitoring of HAND to improve the identification of early stages. Perform fusion analysis of temporal and spatial resolution on HAND at multiple time nodes, accurately locate the location of brain injury, identify how brain injury affects information flow, and reveal potential spatiotemporal markers of HAND. Simultaneous EEG-fMRI provides a method to establish the connection between electrophysiological characteristics and hemodynamic activities and helps to identify future treatment targets for HAND. And use it as the basis for individualized therapeutic intervention to achieve the reversal of HAND, prolong the survival of patients, and improve the quality of life of the patients.
